# Chronic Heart Failure Clinical Practice Guidelines’ Class 1-A Pharmacologic Recommendations: Start-to-End Synergistic Drug Therapy?

**DOI:** 10.7603/s40602-016-0004-5

**Published:** 2016-03-08

**Authors:** Ramon F. Abarquez, Paul Ferdinand M. Reganit, Carmen N. Chungunco, Jean Alcover, Felix Eduardo R. Punzalan, Eugenio B. Reyes, Elleen L. Cunanan

**Affiliations:** Section of Cardiology, Department of Medicine, University of the Philippines, College of Medicine and Philippine General Hospital, 6/F, PGH Compound, Taft Avenue, 1000 Manila, Philippines

**Keywords:** Heart failure, analysis, clinical practice guidelines

## Abstract

**Background:**

Chronic heart failure (HF) disease as an emerging epidemic has a high economic-psycho-social burden, hospitalization, readmission, morbidity and mortality rates despite many clinical practice guidelines’ evidenced-based and consensus driven recommendations that include trials’ initial-baseline data.

**Objective:**

To show that the survival and hospitalization-free event rates in the reviewed chronic HF clinical practice guidelines’ class I-A recommendations as initial HF drug therapy (IDT) is possibly a combination and ‘start-to-end’ synergistic effect of the add-on (‘end’) HF drug therapy (ADT) to the baseline (‘start’) HF drug therapy (BDT).

**Methodology:**

The references cited in the chronic HF clinical practice guidelines of the 2005, 2009, and 2013 American Heart Association/American College of Cardiology (AHA/ACC), the 2006 Heart Failure Society of America (HFSA), and the 2005, 2008, and 2012 European Society of Cardiology (ESC) were reviewed and compared with the respective guidelines’ and other countries’ recommendations.

**Results:**

The BDT using glycosides and diuretics is 79%-100% in the cited HF trials. The survival rates attributed to the BDT (‘start’) is 46%-89% and IDT (‘end’) 61%-92.8%, respectively. The hospitalization-free event rate of the BDT group: 47.1% to 85.3% and IDT group 61.8%-90%, respectively. Thus, the survival and hospitalization-free event rates of the ADT is 0.4%-15% and 4.6% to 14.7%, respectively. The extrapolated BDT survival is 8%-51% based on a 38% estimated natural HF survival rate for the time period109.

**Conclusion:**

The contribution of baseline HF drug therapy (BDT) is relevant in terms of survival and hospitalization-free event rates compared to the HF class 1-A guidelines initial drug therapy recommendations (IDT). Further, the proposed initial HF drug (‘end’) therapy (IDT) has possible synergistic effects with the baseline HF drug (‘start’) therapy (BDT) and is essentially the add on HF drug therapy (ADT) in our analysis. The polypharmacy HF treatment is a synergistic effect due to BDT and ADT.

## Introduction

The prevalence of heart failure (HF) is 1%-2% among adult population in developed countries and 6-10% in the elderly groups. It is rising with an estimated 660,000 new cases each year^1^-^5^. In China, the HF prevalence increased to 29.1% from 16.9%6. The USA HF thirty-day mortality rate has decreased; however, the post-discharge mortality rate, re-admission, and admissions to nursing home facilities have increased. The economic burden of HF remains high^7^-^17^, ^136^-^138^.

A 2004 review has shown that HF disease management programs can reduce HF hospitalizations by 27%. However, HF hospitalization costs in the USA have increased by more than 175% during the last 25 years^18^-^20^. Incomplete implementation of trial methodology, inadequate patient education, absence of trained staff for follow-up monitoring, non-access to specialized HF clinics, application of complex adaptive systems framework, or disease management programs are possible reasons for the continued high burden of HF^21^-^29^. In a systematic review of chronic HF guidelines from Europe, 56% were consensus-based and 28% were evidenced-based advisories^30^-^36^. Furthermore, guidelines recommendations do not highlight the significant contribution of BDT. The concern is the lack of a statement describing that the Class I-A recommended IDT is in fact an ADT to the BDT^44^-^65^.

## Objectives

To determine the survival and hospitalization event free rate in the BDT and IDT groups and to compute for the ADT survival and hospitalization event free rates.

## Methodology

The chronic HF trials published by the 2005, 2009, and 2013 American Heart Association/American College of Cardiology (AHA/ACC), the 2006 Heart Failure Society of America (HFSA), and the 2005, 2008, and 2012 European Society of Cardiology (ESC) were reviewed, summarized, collated, and compared with the guidelines’ class I-A recommendations^38^-^45^. Other chronic HF studies and guidelines were reviewed for comparison^46^-^47^, ^91^-^96^.

BDT refers to the background HF (‘start’) medications used as placebo in the trial. IDT refers to the experimental (‘end’) drug used in the trial and is the guidelines’ suggested first line HF drug therapy. The add-on HF drug therapy or ADT survival and hospitalization event free rate is the absolute value of the difference between the BDT and the IDT rates. The natural HF survival rate of 38% is assumed based on published literature for the time period^107^.

## Results

**Table 1.** Comparison of the 2005, 2009, and 2013 AHA/ACC, HFSA, as well as the 2005, 2008, and 2012 European Society of Cardiology Chronic HF Guidelines Recommendations on Drug Therapy.

Drugs	ACCF/AHA 2005, 2009 &2013	ESC 2005, 2008 and 2012	HFSA 2006
ACE i	• Patients with HFrEF and current or prior symptoms, unless contraindicated, to reduce morbidity and mortality. (I-A) • Used together with a beta blocker. • Same recommendations in 2005 and 2009	• In addition to a beta-blocker, for all patients with an EF ≤40% to reduce the risk of HF hospitalization and the risk of premature death. (I-A) • Same recommendations in 2005 and 2008.	• Routine administration to symptomatic and asymptomatic patients with LVEF <40% (A)
Diuretic	• Patients with HFrEF who have evidence of fluid retention, unless contraindicated, to improve symptoms. (I-C) • (Previously I-A recommendation in 2005 and 2009 guidelines)	• The effects of diuretics on mortality and morbidity have not been studied in patients with HF, unlike ACE inhibitors, beta blockers,and MRAs (and other treatments). However, diuretics relieve dyspnea and edema and are recommended for this reason in patients with signs and symptoms of congestion, irrespective of EF	• Restore and maintain normal volume status in patients with clinical evidence of fluid overload, generally manifested by congestive symptoms (orthopnea, edema, shortness of breath) or signs of elevated filling pressures (A) • Optional for symptomatic treatment
Beta Blocker	• Use of 1 of the 3 beta blockers proven to reduce mortality (e.g., bisoprolol, carvedilol, and sustainedrelease metoprolol succinate) is recommended for all patients with current or prior symptoms of HFrEF, unless contraindicated, to reduce morbidity and mortality. (I-A) • Same recommendation in 2005 and 2009	• In addition to an ACE inhibitor (or ARB if ACE inhibitor not tolerated),for all patients with an EF≤40% to reduce the risk of HF hospitalization and the risk of premature death.(I-A) • Same recommendations in 2005 and 2008	• BB shown to be effective in clinical trials are recommended for patients with EF<40% (A) • Combination of BB and an ACEI is recommended as routine therapy for asymptomatic patients with an LVEF<40% (C) • Majority of patients with LV systolic dysfunction (C)
MRA	Patients with NYHA class II–IV HF and who have LVEF of 35% or less, unless contraindicated, to reduce morbidity and mortality. Patients with NYHA class II HF should have a history of prior cardiovascular hospitalization or elevated plasma natriuretic peptide levels to be considered for aldosterone receptor antagonists. (I-A) • Reduce morbidity and mortality following an acute MI in patients who have LVEF of 40% or less who develop symptoms of HF or who have a history of diabetes mellitus, unless contraindicated. (I-B) • Same recommendation in 2005 and 2009 but more specific laboratory values for monitoring were included	• All patients with persisting symptoms (NYHA class II–IV) and an EF ≤35%, despite treatment with an ACE inhibitor (or an ARB if an ACE inhibitor is not tolerated) and a beta-blocker, to reduce the risk of HF hospitalization and the risk of premature death.(I-A) (Level of Evidence is I-B on 2005 and 2008 guidelines)	• Patients with NYHA Class III/IV, previously Class IV, HF from LV systolic dysfunction (LVEF<35%), while receiving standard therapy, including diuretics (A) • Patients after an acute MI, with clinical HF signs and symptoms and an LVEF<40%. Patients should be on standard therapy, including an ACEI (or ARB) and BB (A)
ARB	• Patients with HFrEF with current or prior symptoms who are ACE inhibitor intolerant, unless contraindicated, to reduce morbidity and mortality (I-A) • Same recommendation in 2005 and 2009	• Reduce the risk of HF hospitalization and the risk of premature death in patients with an EF ≤40%and unable to tolerate an ACE inhibitor because of cough (patients should also receive a beta-blocker and an MRA). (I-A) • Reduce the risk of HF hospitalization in patients with an EF ≤40% and persisting symptoms (NYHA class II–IV) despite treatment with an ACE inhibitor and a betablocker who are unable to tolerate an MRA. (I-A) • Level of Evidence in 2005 and 2008 guidelines is I-B)	• Routine administration to symptomatic and asymptomatic patients with an LVEF<40% who are intolerant to ACEI for reasons other than hyperkalemia or renal insufficiency (A) • Considered as initial therapy rather than ACEI for patients with the following conditions: HF post-MI (A), CHF and systolic dysfunction B) • Routine administration is not recommended in addition to ACEI and BB therapy in patients with recent acute MI and LV dysfunction (A)
Digoxin	• Can be beneficial in patients with HFrEF, unless contraindicated, to decrease hospitalizations for HF. (IIA-B) • Same recommendation in 2005 and 2009	• May be considered to reduce the risk of HF hospitalization in patients in sinus rhythm with an EF ≤45% who are unable to tolerate a beta-blocker (ivabradine is an alternative in patients with a heart rate ≥70 b.p.m.). Patients should also receive an ACE inhibitor (or ARB) and an MRA (or ARB). (IIB-B) • May be considered to reduce the risk of HF hospitalization in patients with an EF ≤45% and persisting symptoms(NYHA class II– IV) despite treatment with a beta-blocker, ACE inhibitor (or ARB), and an MRA (or ARB). (IIB-B)	• Should be considered for patients with LV systolic dysfunction (LVEF<40%) who have signs or symptoms of HF while receiving standard therapy, including ACEI and BB (NYHA II-III [A], NYHA IV [B]) • High dose for the purpose of rate control is recommended (C)

In summary, the chronic HF guidelines recommend the following:

1. ACE i can be given as a routine IDT for systolic dysfunction;
2. ARB is an alternative to ACEi for intolerant symptomatic HF patients;
3. BB is used in all stable patients with systolic dysfunction and chronic HF in addition to ACEI, digitalis, and diuretics;
4. Diuretics is recognized as BDT but HFSA recommends its optional use for symptomatic HF;
5. Aldosterone antagonists (MRA) are add-on to ACEI, BB, digitalis, and diuretics;
6. Digitalis “can be beneficial” as an add-on option in HF in sinus rhythm^36^-^48^

**Table 2.** Survival Rates in the Baseline HF drug therapy (BDT), Initial HF drug therapy (IDT) and Add on HF drug therapy (ADT) Groups in the HF Studies Used in the Reviewed HF Clinical Practice Guidelines.

NAME OF STUDY	DRUGS USED IN THE TRIAL	DRUGS IN BASELINE HF THERAPY	“Baseline HF Therapy” (BDT) (SURVIVAL IN PLACEBO)	“Initial HF Therapy” (IDT) (SURVIVAL IN TRIAL DRUG)	“Add on HF Therapy” (ADT) (SURVIVAL BENEFIT OF TRIAL DRUG)	BASELINE HF THERAPY MENTIONED
**V-HeFT – 1**	Hydralazine + Isosorbide dinitrate	100% on digoxin and diuretics	53.1%	63.8%	10.7%	YES
**SOLVD**	Enalapril	85% on diuretics, 65% on digoxin, 40% on nitrates, 7% on Bblockers	60.3%	64.8%	4.5%	YES
**V-HeFT-2**	Enalapril	60% on vasodilators, 25% on antiarrhythmics	61.8%	67.2%	5.4%	YES
**CONSENSUS**	Enalapril	100% on diuretics, 94% digitalis, 50% vasodilators (mainly nitrates)	46%	61%	15%	YES
**CIBIS II**	Bisoprolol	99% on diuretics, 96% on ACEI or ARB, 58% on nitrates, 51% on digoxin	82.7%	88.2%	5.5%	YES
**MERIT-HF**	Metoprolol CR/XL	>90% on diuretics, >90% on ACEI or ARB, >60% on digitalis	89%	92.8%	3.8%	YES
**COPERNICUS**	Carvedilol	99% on diuretics, 97% on ACEI, 65% on digoxin	81.5%	88.6%	7.1%	YES
**ELITE II**	Losartan	79% on diuretics, 50% on digoxin, 21% on Bblockers, 20% on ACEI	88.3%	89.6%	1.3%	NO (but no benefit)
**CHARM**	Candesartan	85% on diuretics, 55% on B-blockers, 43% on digoxin, 41% on ACEI	75%	78%	3%	YES
**Val-HeFT**	Valsartan	93% on ACEI, 83% on diuretics, 68% on digoxin, 35% on Bblockers	80.3%	80.7%	0.4%	YES
**V-HeFT III**	Felodipine	97% on ACEI, 90% on diuretics, 75% on digoxin	86.2%	87.2%	1%	NO (but no benefit)
**RALES**	Spironolactone	100% on diuretics, 94.5% on ACEI, 74.5% on Digoxin, 10.5% on B-blockers	54%	65%	11%	YES
**EMPHASIS-HF**	Eplerenone	84.3% on diuretics, 78.3% on ACE-1, 19.1% ACE1/ARB, 86.6% B-blockers, 26.6% Digitalis, 14.4% Anti-arrhythmic	84.5%	87.5%	3%	YES
**EVEREST**	Tolvaptan	84.3% ACE1/ARB, 70.8% B-blocker, 97.1% diuretics, 53.6% Aldosterone antagonists	73.7%	74.1%	0.4%	YES
**TOPCAT**	Spironolactone	81% diuretic, 84% ACEI/ARB, 78% beta blocker, 36% CCB, 15% Nitrates, 52% statin	79.6%	81.4%	1.8%	YES

**Legend:** Dig, digoxin; BB, beta-blocker; diu, diuretic; ACEI, angiotensin-converting enzyme inhibitor; ARB, angiotensin receptor blocker; NO, nitrates; Mono, level of monotherapy; **CONSENSUS**, Cooperative North Scandinavian Enalapril Survival Study; **SOLVD**, Studies of Left Ventricular Dysfunction; **V-HeFT**, Vasodilator-Heart Failure Trial; **CIBIS**, Cardiac Insufficiency Bisoprolol Study; **MERITHF**, Metoprolol CR/XL Randomized Intervention Trial in Congestive Heart Failure; US CHF, US Carvedilol Heart Failure Study; **COPERNICUS**, Carvedilol Prospective Randomized Cumulative Survival study; **CHARM**, Candesartan in Heart Failure study; **ELITE**, Evaluation of Losartan in the Elderly trail; **Val-HeFT**, Valsartan Heart Failure Trial; **DIG**, Digoxin Investigation Group trial; **RALES**, Randomized Aldosterone Evaluation Study; **EMPHASIS-HF**, Eplerenone in HFrEF; **EVEREST**, Tolvaptan in acute HF in HFrEF; **TOPCAT**, Spironolactone for HFpEF.

In summary, the reviewed HF studies showed the following:

1. The proportion of HF studies with BDT: 79%-100%
2. The Survival benefit of BDT group: 46%-89%
3. The Survival benefit of IDT group: 61%-92.8%
4. The Survival benefit of ADT group: 0.4%-15%.

**Table 3.** Proportions of Hospitalization and Computed Hospitalization Free Events in the Baseline HF drug therapy (BDT), Initial HF drug therapy (IDT), and Add on HF drug therapy (ADT) Groups in the HF Studies Used in the Reviewed HF Clinical Practice Guidelines (Not hospitalized = 100% – proportion of hospitalized).

Drugs	ACCF/AHA 2005, 2009 &2013	ESC 2005, 2008 and 2012	HFSA 2006
**ACE i**	• Patients with HFrEF and current or prior symptoms, unless contraindicated, to reduce morbidity and mortality. (I-A) • Used together with a beta blocker. • Same recommendations in 2005 and 2009	• In addition to a beta-blocker, for all patients with an EF ≤40% to reduce the risk of HF hospitalization and the risk of premature death. (I-A) • Same recommendations in 2005 and 2008.	• Routine administration to symptomatic and asymptomatic patients with LVEF <40% (A)
**Diuretic**	• Patients with HFrEF who have evidence of fluid retention, unless contraindicated, to improve symptoms. (I-C) • (Previously I-A recommendation in 2005 and 2009 guidelines)	• The effects of diuretics on mortality and morbidity have not been studied in patients with HF, unlike ACE inhibitors, beta blockers,and MRAs (and other treatments). However, diuretics relieve dyspnea and edema and are recommended for this reason in patients with signs and symptoms of congestion, irrespective of EF	• Restore and maintain normal volume status in patients with clinical evidence of fluid overload, generally manifested by congestive symptoms (orthopnea, edema, shortness of breath) or signs of elevated filling pressures (A) • Optional for symptomatic treatment
**Beta Blocker**	• Use of 1 of the 3 beta blockers proven to reduce mortality (e.g., bisoprolol, carvedilol, and sustainedrelease metoprolol succinate) is recommended for all patients with current or prior symptoms of HFrEF, unless contraindicated, to reduce morbidity and mortality. (I-A) • Same recommendation in 2005 and 2009	• In addition to an ACE inhibitor (or ARB if ACE inhibitor not tolerated),for all patients with an EF≤40% to reduce the risk of HF hospitalization and the risk of premature death.(I-A) • Same recommendations in 2005 and 2008	• BB shown to be effective in clinical trials are recommended for patients with EF<40% (A) • Combination of BB and an ACEI is recommended as routine therapy for asymptomatic patients with an LVEF<40% (C) • Majority of patients with LV systolic dysfunction (C)
**MRA**	• Patients with NYHA class II–IV HF and who have LVEF of 35% or less, unless contraindicated, to reduce morbidity and mortality. Patients with NYHA class II HF should have a history of prior cardiovascular hospitalization or elevated plasma	• All patients with persisting symptoms (NYHA class II–IV) and an EF ≤35%, despite treatment with an ACE inhibitor (or an ARB if an ACE inhibitor is not tolerated) and a beta-blocker, to reduce the risk of HF hospitalization and the risk of premature death.(I-A)	• Patients with NYHA Class III/IV, previously Class IV, HF from LV systolic dysfunction (LVEF<35%), while receiving standard therapy, including diuretics (A) • Patients after an acute MI, with clinical HF signs and symptoms and an LVEF<40%. Patients should be on standard therapy,

**Legend:** Dig, digoxin; BB, beta-blocker; diu, diuretic; ACEI, angiotensin-converting enzyme inhibitor; ARB, angiotensin receptor blocker; NO, nitrates; Mono, level of monotherapy; **SOLVD**, Studies of Left Ventricular Dysfunction; **CIBIS**, Cardiac Insufficiency Bisoprolol Study; **MERITHF**, Metoprolol CR/XL Randomized Intervention Trial in Congestive Heart Failure; US CHF, US Carvedilol Heart Failure Study; **COPERNICUS**, Carvedilol Prospective Randomized Cumulative Survival study; **Val-HeFT**, Valsartan Heart Failure Trial; **COMET**, Carvedilol Or Metoprolol European Trial; **RALES**, Randomized Aldosterone Evaluation Study; **CHARM**, Candesartan in Heart Failure study; **EMPHASIS-HF**, Eplerenone in HFrEF; **EVEREST**, Tolvaptan in acute HF in HFrEF; **TOPCAT**, Spironolactone for HFpEF.

In summary:

1. The HF hospitalization free event rate of BDT group: 47.1% to 85.3%
2. The HF hospitalization free event rate of IDT group: 61.8%-90%
3. The HF hospitalization-free event rate of ADT group: 4.6% to 14.7%

p diuretic withdrawal have adverse consequences^175^, ^149^.

education: a systematic review. Srisuk N^1^, Cameron J^2^, Ski CF^3^, Thompson DR.

## Discussion

The chronic HF trials referenced in the chronic HF guidelines listed the use of numerous HF medications which comprised BDT^45^-^48^. The extent of the survival benefit of the BDT is 46%-89% and the IDT is 61%-92.8% with a calculated ADT survival of 0.4%-15%^52^, ^64^-^65^. The extent of the HF hospitalization free event rates of the BDT is 47.1%-85.3% and the IDT drug therapy is 61.8%-90% with a calculated ADT hospitalization free event rate of 4.6%-14.7%^52^, ^64^-^65^. Our review highlights a 6 times (89/15) survival rate in the BDT compared to the ADT and a 6 to 10 times (85.3/14.7 and 47.1/4.6) HF hospitalization- free event rate in the BDT compared to the ADT.

### HF Survival and Hospitalization

Hospitalization marks a fundamental change in the natural history of HF. Three-fourths of all HF hospitalizations are due to symptom exacerbation with one-half of hospitalized HF patients experiencing readmissions within 6 months. Preventing HF hospitalization and re-hospitalization is important to improve patient outcomes and curb health care costs^67^, ^68^.

Avoidance of hospital admission can be equivalent to prolonging quality of life^61^-^71^.

Repeat HF hospitalization ranged from 22.7% in Latin America and 43.9% in North America^143^-^144^. Two-thirds of patients hospitalized for acute decompensated chronic heart failure have already survived a known history of heart failure.^145^-^146^. In the OPTIMIZE-HF Registry, rates of re-hospitalization were 30% post discharge^173^-^174^. In the EVEREST trial, 40% of post discharge deaths were from HF^173^-^174^. A prior history of HF decompensation or hospitalization identifies patients who are particularly at high risk of recurrent events^147^-^148^. Is HF re-hospitalization associated with ADT with or without BDT?

### Baseline HF Drug Therapy

**Withdrawal effect:** A meta-analysis of loop diuretics in HF found a statistically significant survival benefit on top of baseline HF therapy^74^. Studies have showed that ACEi or digoxin use lowered mortality (OR 0.24); reduced worsening HF (OR 0.07), and improved exercise capacity. (OR 0.72)^72^-^75^. The PROVED and RADIANCE showed worsening HF occurred at 4.7% (digoxin, ACEi and diuretic therapy); 25% (ACEi and diuretic therapy); and 39% on diuretic alone (76-83) after withdrawal. Thus, the combination of digoxin, ACEi, and loop diuretic are relevant as BDT. Digoxin and loop diuretic withdrawal have adverse consequences.^175^, ^149^.

**Diuretic effect:** Doubling the dose of diuretics among symptomatic HF patients on beta blockers, ACEi or ARB, spironolactone, and digoxin, led to significant loss of weight, improvement in symptoms, and an increase in 6-minute walk distance^150^-^151^. Is this a cardio-renal effect?

**Digoxin use and level:** Recent opinions say that “not enough data supports the use of digoxin with current medications for chronic systolic heart failure like betablocker, spironolactone, and ACEI.” Thereby, the use of digoxin has not been emphasized in chronic heart failure treatment” guidelines^152^. There is evidence to show the contrary.

One study showed a 34% lower rate of all cause hospital admission in patients assigned to digoxin. This finding highlights an early beneficial effect of digoxin. 44% of patients enrolled in the DIG study used digoxin, those on digoxin maintained the treatment, while digoxin was stopped (without a washout period) among those assigned in the placebo153. Can this explain why the DIG study did not show all-cause mortality reduction since the placebo arm previously benefited from digoxin use?

Other studies have shown all-cause hospitalizations occurred in 5.4% vs 8.1% among chronic HF patients on the digoxin and placebo groups, respectively, (HR= 0.66). The 30-day cardiovascular hospitalization (HR 0.53; 0.38-0.72) and heart failure hospitalization (HR 0.40; 0.26-0.62) favored the digoxin group with similar trend in all-cause mortality (HR 0.55; 0.27-1.11). Younger patients were at lower risk of events and obtained similar benefits from digoxin154. Further, digoxin was associated with long-term improvement in kidney function and reduction in death or hospitalization^157^-^159^, ^91^-^94^, ^100^.

Digoxin reduces hospitalizations and improves symptoms when dosed to achieve low serum concentrations of 0.5-0.9 ng/ml (HR 0.81; 0.71–0.92)^176^. Further, studies have showed that lower serum digoxin concentration (0.5–0.9 ng/mL) was associated with reduced all-cause mortality (HR 0.77; 0.67–0.89), cardiovascular mortality (HR 0.83; 0.71–0.97), and heart failure mortality (HR 0.63; 0.49–0.82) (162). Current guidelines do not sufficiently emphasize the need to achieve low serum digoxin concentrations^160^-^161^. The DIG study is the only chronic HF study with serum digoxin level (SDL) determination.

### Add on HF Drug Therapy

Total mortality or hospitalization, MI, and stroke did not differ between ARB and ACEi. Adverse effects resulted in increased withdrawals with combination ACEi and ARB^101^. Studies on BB therapy showed it improved survival, hospitalization, LV function, dyspnea, exercise tolerance time, NYHA FC, reduced death or readmission (OR=0.74), death or re-infarction (OR=0.77) or sudden death (OR=0.80)^102^, ^103^, ^163^, ^164^.

Short-term effects of BB withdrawal in acute decompensated heart failure have been reported^177^. BB withdrawal significantly increased risk of in-hospital mortality (RR 3.72;1.51 to 9.14), short-term mortality (RR 1.61;1.04 to 2.49), and combined short-term rehospitalization or death (RR1.59; 95% CI: 1.03 to 2.45). This data suggests BB should be continued in HF patients unless contraindicated^165^.

In CAD patients with heart failure and preserved systolic function, low-dose digoxin was significantly more effective than ivabradine^166^. Digitalis showed an OR for mortality of 0.98 (0.89-1.09), hospitalization of 0.68 (0.61-0.75), and clinical HF deterioration of 0.31 (0.21-0.43). Digoxin has no effect on long-term mortality; however, it reduced hospitalization and improved clinical status of symptomatic HF patients^104^, ^105^.

### The Extrapolation

In the 21^st^ century, the combination use of ACEI, ARB, BB, and aldosterone antagonist decreased hospitalizations and improved survival^112^-^113^. However, baseline HF drug therapy with digoxin and diuretics is a relevant concern^66^,^48^, ^114^.

If the recommended initial HF drug therapy survival rate is actually the add-on HF drug therapy recommended Class 1-A survival rate (computed as initial HF drug therapy survival rate MINUS the baseline HF drug therapy survival rate), then the computed add on HF drug therapy survival rate will be 0.4%-15%. Similarly, the computed add on HF drug therapy hospitalization free event rate will be 4.6%-14.7%.

The natural HF history survival in five years prior to current evidenced-based effective therapy is assumed to be 38%107. Therefore, given the derived baseline HF drug therapy survival rate of 46% to 89% MINUS 38% assumed natural HF survival rate, the extrapolated baseline HF drug therapy survival rate is 8% to 51% which is higher than the add-on HF drug therapy Class 1-A recommendation survival rate of 0.4%-15%.

### Economic Impact of HF treatment

“The implementation of evidence-based therapy for HF treatment is not only clinically efficacious, but also economically attractive”^97^. To implement cost-effective strategies and contain the HF hospitalization epidemic, optimal identification of high-risk individuals and various multi-marker risk prediction schemes have to be developed^98^. Indeed, digoxin use gave a cost saving of >50% of several higher-risk HF patient subgroups^99^. Thus, combination HF therapy is still related to cost and clinical benefits.

## Guidelines Adherence

In chronic HF cases and despite management by cardiologists, medical prescription differed substantially (> 50%) from guidelines’ recommendations^167^. The percentage of patients taking β-blockers was 38%; the percentage taking angiotensin-converting enzyme inhibitors/angiotensin receptor blockers (ACEI/ARBs) was 32%^168^. Target doses for ACEi or ARB and BB were low at 40.3% and 28.9%, respectively^169^. Furthermore, the Heart Failure Adherence and Retention Trial has determined that 37% did not adhere to HF evidence-based guidelines^170^. In the China Outpatient HF Study, patients received target dose of ACEI/ARB (17.92%), BB (17.92%), respectively^171^. The low guideline directed medical therapy, usually IDT adherence, highlights the relevance of BDT

## Limitations

The HF studies reviewed were limited to references and our analysis depended on the published trial data cited in the AHA/ACC, HFSA, and the ESC chronic HF guidelines without uniform “chronic HF definitions” although “unstable HF state” was excluded^38^, ^39^, ^41^-^45^. A later guideline review classified HF with typical HF symptoms, physical findings and definitive EF levels^46^.

The specific value of BDT and ADT to HF natural disease progression are unclear and hard to quantify at present. Whether digoxin added cost savings and reduced mortality and hospitalization is also speculative at this time. Other issues may affect the HF natural survival history thereby reducing the extrapolated survival benefits attributed to the BDT such as the following: (i) the contribution of renal failure, respiratory disease, anemia, cognitive impairment, falls and urinary incontinence^118^; (ii) the ‘real world’ acute HF exacerbations and re-admissions mortality of 8.2% that is independent of age, BP and creatinine levels^119^, ^120^; (iii) the 9.6% mortality and 19.4% re-hospitalization for CV causes at 90 days of HF admission^121^; (iv) the transition from preserved EF to reduced EF or a mixture of both^122^; (v) the higher cost of different HF diagnostic and management options^123^; (vi) the inability or poor utilization of HF biomarkers due to cost^124^; (vii) the adaptation of HF clinical pathways125; (viii) the presence of psycho-socioeconomic factors that are independent of HF development and leads to adverse outcomes^126^, (ix) the interactions between multiple drugs which affects acceptance and compliance^127^, and (x) family education at home to enhance patient self-care, boost dietary and treatment adherence^182^.

These undetermined and still unrecognized factors impact on the natural HF history and were not analyzed in this paper. Whether digoxin added cost savings and reduced mortality and hospitalizations can translate into substantial changes in the survival benefit attributable to baseline therapy is also speculative at this time.

## Conclusion

The contribution of baseline HF drug therapy (BDT) is relevant in terms of the survival and hospitalization-free event rates compared to the HF class 1-A guidelines recommendations (IDT). Further, the proposed initial HF drug (‘end’) therapy (IDT) has possible synergistic effects to the baseline HF drug (‘start’) therapy (BDT) and is essentially the add on HF drug therapy (ADT) in our analysis. The polypharmacy HF treatment is a synergistic effect due to BDT and ADT.
